# Chaste: An Open Source C++ Library for Computational Physiology and Biology

**DOI:** 10.1371/journal.pcbi.1002970

**Published:** 2013-03-14

**Authors:** Gary R. Mirams, Christopher J. Arthurs, Miguel O. Bernabeu, Rafel Bordas, Jonathan Cooper, Alberto Corrias, Yohan Davit, Sara-Jane Dunn, Alexander G. Fletcher, Daniel G. Harvey, Megan E. Marsh, James M. Osborne, Pras Pathmanathan, Joe Pitt-Francis, James Southern, Nejib Zemzemi, David J. Gavaghan

**Affiliations:** 1 Computational Biology, Department of Computer Science, University of Oxford, Oxford, United Kingdom; 2 CoMPLEX, Maths & Physical Sciences, University College London, London, United Kingdom; 3 Centre for Computational Science, University College London, London, United Kingdom; 4 Department of Bioengineering, National University of Singapore, Singapore, Singapore; 5 Oxford Centre for Collaborative Applied Mathematics, Mathematical Institute, University of Oxford, Oxford, United Kingdom; 6 Computational Science Laboratory, Microsoft Research, Cambridge, United Kingdom; 7 Centre for Mathematical Biology, Mathematical Institute, University of Oxford, Oxford, United Kingdom; 8 Department of Mathematics and Statistics, University of Saskatchewan, Saskatoon, Canada; 9 Food and Drug Administration, Silver Spring, Maryland, United States of America; 10 Fujitsu Laboratories of Europe, Hayes Park, London, United Kingdom; 11 CARMEN project, INRIA Bordeaux Sud-Ouest, Talence, France; UCSD, United States of America

## Abstract

Chaste — **C**ancer, **H**eart **A**nd **S**oft **T**issue **E**nvironment — is an open source C++ library for the computational simulation of mathematical models developed for physiology and biology. Code development has been driven by two initial applications: cardiac electrophysiology and cancer development. A large number of cardiac electrophysiology studies have been enabled and performed, including high-performance computational investigations of defibrillation on realistic human cardiac geometries. New models for the initiation and growth of tumours have been developed. In particular, cell-based simulations have provided novel insight into the role of stem cells in the colorectal crypt. Chaste is constantly evolving and is now being applied to a far wider range of problems. The code provides modules for handling common scientific computing components, such as meshes and solvers for ordinary and partial differential equations (ODEs/PDEs). Re-use of these components avoids the need for researchers to ‘re-invent the wheel’ with each new project, accelerating the rate of progress in new applications. Chaste is developed using industrially-derived techniques, in particular test-driven development, to ensure code quality, re-use and reliability. In this article we provide examples that illustrate the types of problems Chaste can be used to solve, which can be run on a desktop computer. We highlight some scientific studies that have used or are using Chaste, and the insights they have provided. The source code, both for specific releases and the development version, is available to download under an open source Berkeley Software Distribution (BSD) licence at http://www.cs.ox.ac.uk/chaste, together with details of a mailing list and links to documentation and tutorials.

This is a *PLOS Computational Biology* Software Article

## Introduction

**C**ancer, **H**eart **A**nd **S**oft **T**issue **E**nvironment (Chaste) has been developed to enable the study of novel problems in computational physiology and biology. The following quotation from a recent article by Wilson highlights two problems that Chaste has been designed to overcome:

“Increasingly, the real limit on what computational scientists can accomplish is how quickly and reliably they can translate their ideas into working code.” [1]

**First**, the *speed* at which progress can be made by researchers in our field is typically limited because previously developed models and methods are often not re-used effectively. At the most practical level, model equations and algorithms should be encoded as software (or, more usefully, as mark-up languages for generating software [2]), describing unambiguously the computations required for simulations. In computational physiology and biology, many problems share a need for the same underlying components and numerical schemes. It is still common for each new PhD student or post-doctoral researcher to ‘re-invent the wheel’ and develop, for example, their own mesh structures, ordinary/partial differential equation (ODE/PDE) solvers and input/output (IO) interfaces. This not only slows progress, but a lack of formal software training in structuring and documenting code can lead to code that is difficult to follow and untangle (known as ‘spaghetti code’) [3]. Such code rapidly becomes unusable by anyone else, and is typically discarded at the end of a project, requiring the next person to work on the research topic to start the process again.

**Second**, the *reliability* of code, and subsequent results, is often uncertain and unprovable. As discussed by Baxter *et al.* in a perspective on software development in this journal [4], there is generally no rigorous software testing approach taken, and testing comes down to whether results ‘look about right’ [3]. This may soon become safety-critical, as clinical interventions become guided by the results of computational biology simulations.

The problems discussed above lead to it being very difficult, if not impossible, to guarantee the *reproducibility* of computational results. Minimum information standards have been suggested for models (MIRIAM [5]) and simulations (MIASE [6]), defining vital requirements underpinning reuse and reproducibility. Mark-up languages such as SBML [7], CellML [8], FieldML [9] and SED-ML [10] help to satisfy these requirements in a machine-readable format. Given the complexity of modern mathematical models and numerical algorithms, we believe the use of such standards in open source software is a pre-requisite for *rapid progress*, *reliability* and *reproducibility*.

To date, our primary applications have been in computational physiology and biophysics. In these fields, a wide array of models are represented as continuum ODE/PDE problems, individual or agent-based discrete models, or a hybrid of these two. Examples of problems falling into these categories include cardiac electrophysiology and electromechanics, tumour growth, and developmental biology. In 2005, we began to build Chaste as a software environment that could be used for simulation of these types of problem, which would overcome many of the pitfalls discussed above. Most commercial software is closed source and difficult to extend to study novel models, as these may include a different class of equation or completely different modelling paradigm. In the limited cases where free open source software was available for our applications, we frequently found it difficult to test and extend. We wanted to create a software library that could rapidly evolve, to keep pace with our scientific investigations. To achieve this, Chaste comprises a library of fully-tested modules for the common elements of our application areas , which can be easily utilised and readily extended to the simulation of novel models, and to the use of novel numerical algorithms. We believe Chaste is a good example of software that follows the ten simple rules for open development of scientific software [11].

Other notable open source codes have been developed, including OpenCMISS and Continuity for continuum modelling [12], [13], CompuCell3D for cellular Potts modelling [14] and MultiCellXML for agent-based modelling and simulation data [15]. However, Chaste is the only open source software available for many of its application areas, and is exceptional in that industrial software engineering standards have been used for its development. Chaste is also unique in being well suited for both continuum and discrete modelling approaches, and is well-positioned for the investigation of hybrid approaches [16].

Release 1.0 of Chaste occurred in 2009 and has been described previously [17], [18]. Through the use of examples, we will describe the capabilities of the newly-released version 3.1, novel scientific applications, and future directions. It is our intention that all of the examples in this article can be reproduced on a desktop PC; they are therefore less computationally intensive than many of the simulations performed in scientific research. The example simulations can be run, and figures recreated, by downloading the associated Chaste project from http://www.cs.ox.ac.uk/chaste/download, as described in Text S1.

## Design and Implementation

In this section, we discuss the Chaste development strategy, as this is fundamental to its properties, capabilities and extensibility for novel problems. We then introduce the code layout and the available model types and algorithms. Chaste is written in C++, a compiled language that allows object-oriented class definitions. This makes the code suitable for applications where efficient memory management and performance are key, but also allows simple extension and inheritance of existing functionality.

At present Chaste can only be used with Linux, although it works well via a Linux virtual machine (using software such as VirtualBox) on a host running Microsoft Windows or Mac OS X. The code needs to be compiled after downloading and setting up dependencies. For users (rather than developers modifying Chaste itself) this is a one-off event. Due to the volume of source and test code, compilation can take considerable time, particularly for an optimised build, on the order of hours using a single processor, or around 30 minutes on an 8-core server.

### Development Strategy

As far as we are aware, Chaste is the only code of its kind that has been developed using agile and test-driven development. We believe that our experience of this development style has valuable insights for other research groups, highlighted in this section. We have found the following practices invaluable in terms of rapid development, delivering high performance, and ensuring reliable results. An independent analysis of Chaste development activity and code composition can be found at Ohloh (http://www.ohloh.net/p/Chaste) .

#### Test-driven development

Test-driven development is fundamental to our efforts. In this style of development, ‘test code’ is written *before* the ‘source code’ which will actually perform the function we require. Once a test is in place, the source code is then written to make the test pass. This has the advantage of forcing developers to consider the best interface for their new code, and to consider how to test that the source code performs its function correctly. There are then two discrete collections of code in each module of Chaste. The ‘test’ code makes use of the ‘source’, as if the source code were any other C++ library, and checks that it performs as intended. The ‘source’ never makes use of ‘test’, and only ‘source’ is compiled into a library for use in other modules (and third party programs).

The test code is uploaded (‘committed’) to the central version control repository along with the source code that it tests. Upon each commit, all the tests are run in order to check that no functionality has been inadvertently broken (‘continuous integration’). This ensures the code always performs as intended, and developers ‘protect’ their code from any future changes to either the code itself, or any code it relies on. This approach does not guarantee bug-free code, but in practice makes bugs very rare. When bugs do occur, this is typically because functionality is expected that has not been fully tested, and the first step in the solution is to write more tests.

Additional tests are run each night, which: check all of the standard tests for memory leaks; profile the speed of different parts of the code ; check for documentation on all source code; and check that every line of the source code is executed by at least one of the tests (‘coverage’). Among all the coding practices we use, *test-driven development* is never abandoned, and is the feature most highly regarded by the development team, who commonly apply it to their other projects.

#### Agile programming

Chaste is developed using an ‘agile’ development methodology, using many features of eXtreme Programming (XP) [19]. One aspect of this approach is to avoid planning too far ahead at any stage. This limits the scope of coding work at any time to a goal that is achievable in a reasonable time frame (typically one month). This approach allows the fast development of working prototypes, and removes ‘paralysis through planning’, which can occur when trying to accommodate a myriad of possible future requirements. However, significant time is spent re-working existing code: class structures and interfaces are reorganised for efficiency, readability and ease of re-use. Overall, this approach generates effective code over time and flexibility is added as required.

We have also adopted some other characteristics of XP, notably ‘pair programming’. Ideally, all contributed code is written by a pair of developers, sitting side by side, with one writing code, and the other checking and suggesting improvements. In an academic setting, we have found that this need not be insisted upon, but we use it in regular coding sessions. A particular benefit in an academic setting, where people may move on to new projects frequently, is that no single person takes sole responsibility for any part of the code. Simple rules are adhered to for the naming of variables, methods and classes, which enables developers , and new users, to navigate their way through the code efficiently, and makes mistakes less likely. For further details please refer to our developers' wiki: https://chaste.cs.ox.ac.uk/trac/wiki/GettingStarted.

### Code Layout and Design

Chaste provides libraries for code which is common to many computational biology problems. Here we briefly describe the components of the code and their capabilities. We will present and discuss example simulations, and the new scientific insight Chaste has enabled, in the Results section.

- global — contains code for basic mathematics (including a random number generator), time stepping, checkpointing (saving and loading simulations) using the Boost serialization library [20], parallel vector classes, and code to handle warnings and errors.
- io (input/output) — code for reading, writing and conversion of various file formats, including modules to handle the HDF5 scientific file format [21], which enables distributed data to be collated and stored in a single file.
- mesh — code for linear or quadratic tetrahedral meshes and vertex meshes; nodes, elements, boundary properties; mesh generation; mesh distribution using METIS/parMETIS [22]; readers and writers for Triangle/TetGen [23], [24], Meshalyzer, Cmgui (http://www.cmiss.org/cmgui) and VTK (Paraview) [25] formats.
- linalg (linear algebra) — code which uses Boost uBLAS [20] and PETSc [26] for vector and matrix operations.
- ode — code for defining ODEs; solvers, basic finite difference schemes, the Sundials CVODE solver [27]; termination on root-finding capabilities.
- pde — code for defining elliptic and parabolic second-order PDEs; parallel finite element solvers of generic coupled systems of PDEs (using mesh and linalg).
- continuum mechanics — code for solving compressible and incompressible general non-linear elasticity problems.

These core components are used by two main application components that have driven Chaste development to date — cell based and heart, discussed in the Results section.

#### Projects

‘Bolt-on’ projects for individual studies using Chaste are supported via an interface to the build infrastructure. This also provides a link to our framework for test-driven development of code. Continuous testing can be utilised to ensure that your code maintains the capability to reproduce old results as it evolves. If common code occurs in more than one project, we encourage developers to migrate this code to the central Chaste repository, thus making it publicly available. Access to a project subversion repository hosted at Oxford, and integration into the Chaste wiki, is available for academic researchers upon request. This enables all users to release a project with each publication, allowing all of the results and figures in an article to be reproduced with a given version of Chaste. The following references are to papers associated with bolt-on projects that are freely available to download, reproduce, adapt and extend [17], [28]–[31]. Key scientific findings arising from these papers are discussed in the Results section below. See Text S1 for instructions on downloading the project associated with this article.

## Results

Here we provide some examples of the types of scientific problems that Chaste has been used to investigate to date. This section is structured around four examples, which can all be reproduced by running the tests in the project accompanying this paper. Each example is simple, so as to enable reproduction on a typical desktop PC. The code for each example is presented in a tutorial-style wiki pages at https://chaste.cs.ox.ac.uk/trac/wiki/PaperTutorials/Plos2013. Details of visualization packages used to create the figures are contained on these wiki pages. Primary support is for the open source VTK visualization file format, although other formats can be output for specific types of simulation (for example the cardiac component also provides Cmgui and Meshalyzer formats).

### Cell-based

The cell_based code provides a range of modelling frameworks for individual-cell-based simulations. Common code is provided for cell-cycle models, cell death events, intracellular signalling pathways, and for coupling to PDEs, e.g. for the diffusion of nutrients or oxygen. For cellular simulations, there are two main types of model: on- and off-lattice.

**On-lattice** models which are supported include cellular automata (1–3D) [32], in which each lattice site is associated with one or more cells. Advanced models of angiogenesis (the growth of new blood vessels from pre-existing vessels) have been built using the cellular automata framework [33]. Cellular Potts models are also available (1–3D), in which a cell occupies a number of lattice sites [14], [34], and additional sites are included or removed to minimize an energy function.

**Off-lattice** models fall into two main types, with cells being defined spatially by (i) their centres, or (ii) their vertices:

- In cell-centre models cells are represented by points, which move according to interactions with neighbouring cells that can be defined in two main ways: node-based (1–3D), where neighbours are any nodes within a certain interaction distance; or mesh-based (1–3D), where neighbours are nodes which share elements of a mesh (defined by a Delaunay triangulation of the cell centres).
- Vertex dynamics models (2D) represent cells as polygons whose vertices move in response to forces. In some vertex dynamics models, a free energy function is specified, whose gradient is assumed to exert a force on each vertex [35]. Elsewhere, the forces acting on each vertex are provided explicitly [36]. An extension of vertex models to 3D is planned for future work.

We have used Chaste to examine the suitability of these different modelling paradigms for the same biological problem, highlighting idiosyncrasies [16], [37].

**Figure 1:**
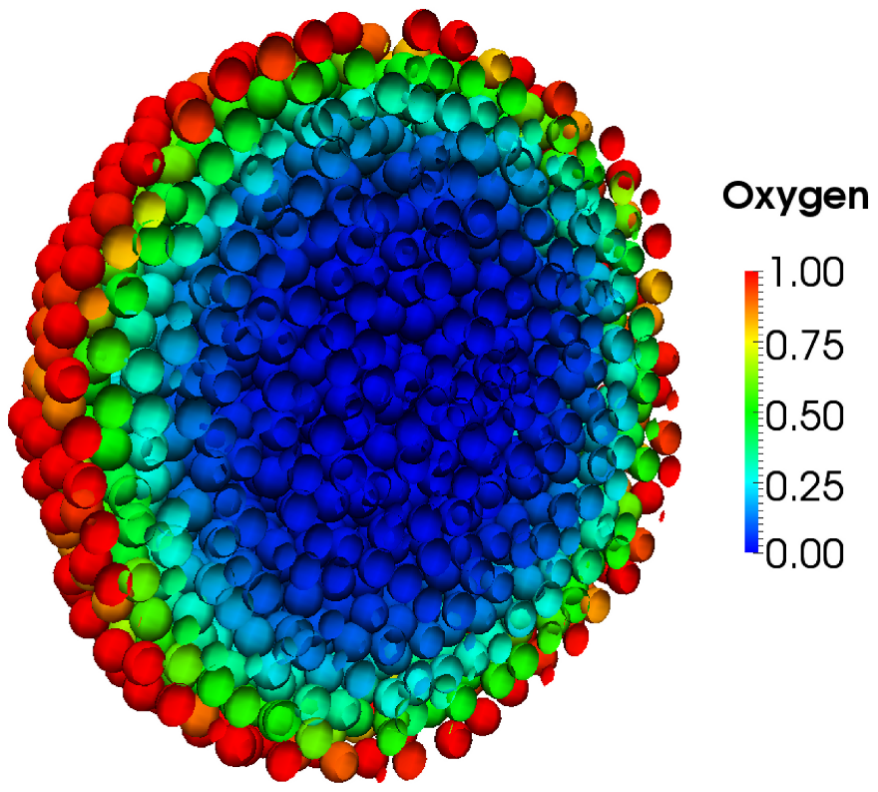
3D off-lattice simulation coupled to PDE: 3D simulation of a tumour spheroid. A cross-section of a tumour spheroid is presented. Cell centres, nodes of a mesh, are represented by spherical shells and coloured according to the local oxygen concentration. Proliferation is dependent on oxygen, which diffuses and is taken up by cells in the spheroid, such that only cells near the outer rim divide. Cell death occurs under low oxygen conditions near the centre of the spheroid. See also Video S1.

Our first example uses an off-lattice mesh based 3D simulation, coupled to a PDE , to simulate the diffusion and consumption of oxygen within a growing tumour spheroid . The PDE boundary condition is a fixed oxygen concentration at the spheroid surface; oxygen diffuses and is taken up by cells. In low oxygen conditions cells cease to proliferate, and in very low conditions in the centre of the spheroid they die (see Figure 1 and video S1), as observed in experiment.

Our second example is taken from the crypt code component, developed to study intestinal crypts and the initiation of colorectal cancer. This component includes code to define the intestinal crypt geometry, Wnt signalling pathway and intestinal cell-cycle models. In van Leeuwen *et al.* [28] Chaste was used to predict monoclonal conversion in the colorectal crypt as a result of simple competition arising from the mechanics of the stem cell population. This prediction has since been confirmed experimentally [38], [39]. We have used the Chaste off-lattice mesh-based simulations extensively to examine the concept and role of stem cells in crypt homeostasis [31], [40], as well as the contribution of mechanical effects to cell behaviour [41], [42]. A hypothesis generated following Chaste simulations related to cellular extrusion in the crypt [42] was verified experimentally in independent investigations, published at a similar time [43].

**Figure 2:**
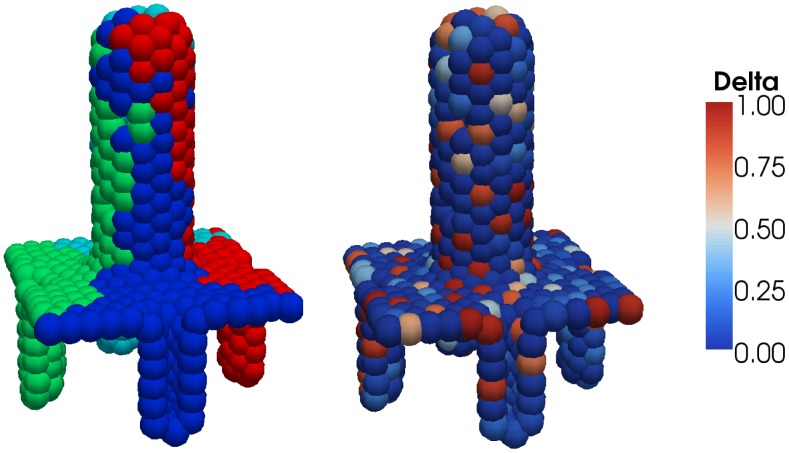
3D off-lattice simulation confined to a 2D surface: small intestinal crypts and villus. Left: cells are labelled according to their ancestor cell; each crypt gives rise to a monoclonal population, with a multiclonal villus comprised of cells from each crypt. Right: the same simulation, here with cells labelled according to Delta levels (non-dimensionalised); Delta-Notch patterning occurs due to a signalling model inside each cell, which depends on the activity of neighbouring cells, and is thought to lead to differentiation into secretory and absorbative cell types. See also Video S2.

In Figure 2 we present an example that is also based on the intestinal epithelium: a 3D off-lattice node-based simulation confined to a 2D surface. In the small intestine, finger-like projections, called villi, are surrounded by a cluster of crypts. In our simulation, four crypts are considered, and cells move according to a nearest-neighbour-defined repulsive force.

Over the course of the simulation each of the four crypts becomes monoclonal, leading to a villus carrying each of the four clonal populations (see video S2). In addition, each cell is carrying an ODE system for Delta-Notch signalling, which takes as an input the average Delta level of the neighbours [44]. This leads to a dynamic pattern of Delta-Notch with high/low activity in neighbouring cells, thought to lead to differentiation into secretory and absorbative cells [45].

### Heart

The heart component of Chaste provides fast and accurate solution of electrophysiological problems on large meshes, optimised for high-performance computing facilities by using PETSc for parallel linear algebra, and (par)METIS for mesh distribution [17], [46].

Simulations can be performed on single-cell ODE systems, or at the tissue/whole-organ level using PDE formulations such as monodomain, bidomain, bidomain-with-bath, and our new extended-bidomain system [47]. Spatial heterogeneities in fibre directions and cardiac cell models (and their parameters) can be included; and post-processing can be performed to obtain quantities such as action potential durations, pseudo-ECGs [48], and conduction velocities.

**Figure 3:**
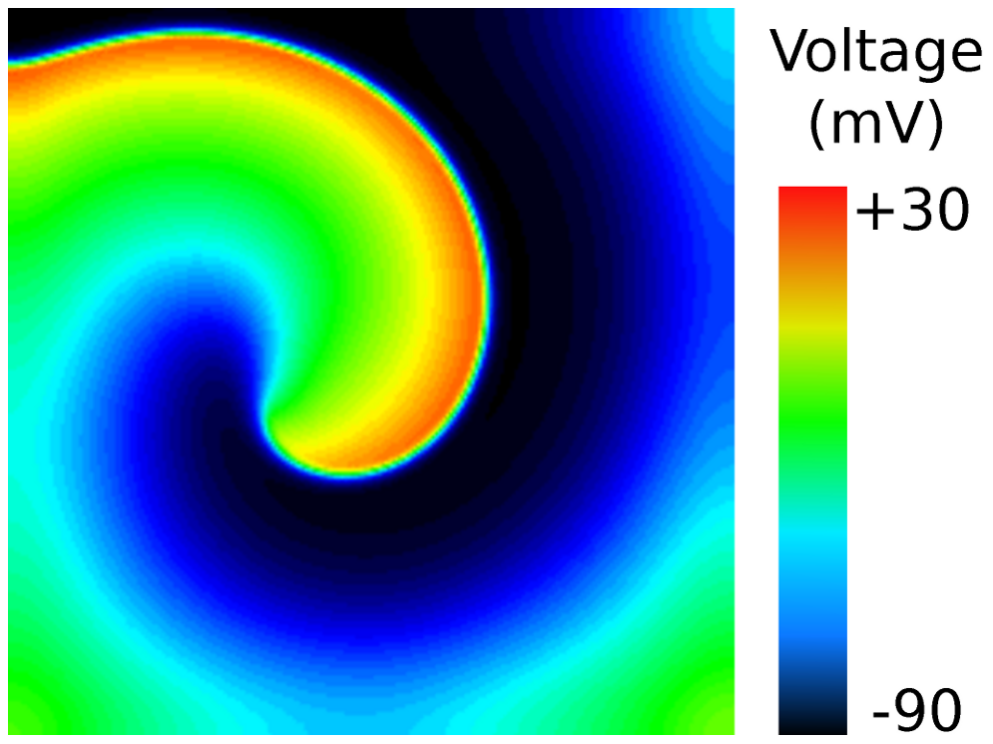
Cardiac electrophysiology: a re-entrant spiral wave. This figure displays the membrane voltage in a 2-D 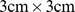 monodomain simulation using the Luo-Rudy 1991 action-potential model [50] with the modifications and protocol suggested in [49]. See also Video S3.

The behaviour of electrical waves in cardiac tissue is commonly studied to understand how changes to ion-channel dynamics, through disease or drug block, can lead to the onset of fatal arrhythmias. In Figure 3 we present a snapshot of a 2-D monodomain simulation which reproduces one of the results of a study of electrical wave dynamics [49] (see video S3). A stable spiral wave is generated on a 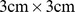 mesh, by appropriate stimulation and alteration of ion-channel expression in a cardiac action potential model [50].

The development of a mesh of the human heart embedded within a torso enabled us to simulate the 12-lead human body surface ECG, and to predict ECG changes under drug action [51]. These techniques have the potential to be used to predict the results of human clinical trials during drug development, to improve the attrition rates in drug development, and to help prevent dangerous drugs reaching the market [30], [52], [53] . Simulations have also been performed to study defibrillation of a human heart in arrhythmia [54], with a view to improving medical devices and interventions.

Special capabilities have been introduced to handle CellML files (cardiac cell model definitions), including automatic units conversion and run-time compilation (dynamic loading) [55]. These features have enabled studies that consider the extensive variability between different models [29], [30], [56]. Our group has also used Chaste to study the effect of stochasticity in cardiac models [57]–[59], a cause of variability in experimental recordings, and potentially linked to pro-arrhythmic risk.

Improvements to the numerical algorithms used in cardiac simulation have been a large focus of our research efforts, and subsequent speed improvements have enabled the novel biological problems above to be studied. We have focussed on efficient solution of action potential models [55], [60], [61], matrix preconditioners [62], [63], adaptivity [64], and choice of finite element formulation [56], [65], [66].

Chaste displayed exceptional performance in terms of accuracy and convergence properties in a recent independent study surveying major cardiac electro-physiology solvers [67], and its modular nature allowed us to explain the behaviour of other solvers [68]. Independent benchmarking has shown good scaling to 2048 cores [69], with further improvements recently [70].

**Figure 4:**
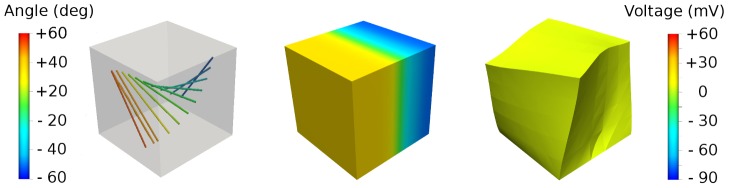
Cardiac electromechanics in a ventricular wedge: left, fibre orientation relative to 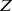 axis; middle and right, simulation of electrical propagation and deformation (middle: 1 ms after stimulus on left face (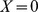); right: 35 ms after stimulus). See also Video S4.

We have also begun to work on electromechanics [71], this becoming publicly available from v3.0 onwards. In electromechanical models, cellular tension develops in response to electrical activation and calcium release. The tension informs a model of mechanical properties of cardiac tissue, governing its deformation. The forces that the tissue experiences can then trigger stretch-dependent electrical activity, forming a feedback system. We use this electromechanics code for our final example in Figure 4. Figure 4 shows varying fibre directions throughout a 3-D block of tissue, alongside the spread of an electrical wave, and subsequent tissue deformation (see also video S4).

### Other Application Areas

The Chaste library has found applications in other areas of computational biology. Over the last decade, there has been a strong interest in building a multi-scale modelling framework to study the contractile function of the human gastrointestinal system [72], [73]. The authors have used Chaste to integrate the detailed cellular electrophysiology of the stomach into a whole organ framework with a view to studying the effects of genetic mutations as well as diseases such as diabetes [47], [74].

A number of other groups are using Chaste for a large variety of simulations. The following groups have given us their permission to describe their applications of Chaste: lattice-based multiple occupancy cellular automata (Markus Owen, University of Nottingham); the effects of radiation on tissue (Shaowen Hu, NASA); sexually transmitted infection modelling (Martin Nelson, University of Nottingham); cardiac electrophysiological modelling (US Food and Drug Administration); drug-induced changes to cardiac rhythm (Safety Pharmacology, GlaxoSmithKline); and neuron electrophysiology (Andres Agudelo-Toro, Max Planck Institute University of Göttingen).

## Availability and Future Directions

Chaste is freely available to download from the website at http://www.cs.ox.ac.uk/chaste. Releases 1.0–3.0 were under the LGPL v2.0 licence, while v3.1 and future releases are under the more flexible 3-clause BSD licence, to facilitate use of the code by industrial partners.

Currently, we are adding support for the following types of simulation: parallel cardiac electro-mechanics; His-Purkinje system in cardiac electrophysiology; lung mechanics; import of SBML models for signalling pathway and cell-cycle models; fluid flow for haemodynamics and airflow. We are investigating ways to make it easier for users to contribute code, and aim to support a Microsoft Windows environment in the next year.

### How to Become an Active Developer

From the main Chaste website you can sign up to the users' mailing list to receive announcements of new versions, and support from users and developers. You can also register to create a login for the Chaste wiki to view and comment on work tickets, and submit patches for inclusion in Chaste. In addition to the examples associated with this article, we have a large number of tutorials that are easy to adapt to perform many types of simulation. There is a guide for people who would like to work with the latest development version of Chaste at:

https://chaste.cs.ox.ac.uk/trac/wiki/ChasteGuides/ExternalDeveloperGuide.

## Supporting Information

Software S1 **A Zip file containing the Chaste project that forms the supplementary material, it can be used to recreate the figures in this article.** This project is compatible with Chaste 3.1 only.
(ZIP)

Text S1 **Further details on installation of Chaste and dependencies.**
(PDF)

Video S1
**3D off-lattice simulation coupled to PDE: 3D simulation of a tumour spheroid.** A cross-section of a tumour spheroid is presented. Cell centres, nodes of a mesh, are represented by spherical shells and coloured according to the local oxygen concentration. Proliferation is dependent on oxygen, which diffuses and is taken up by cells in the spheroid, such that only cells near the outer rim divide. Cell death occurs under low oxygen conditions near the centre of the spheroid. Shown from 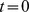 to 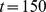 hours.(MP4)

Video S2
**3D off-lattice simulation confined to a 2D surface: small intestinal crypts and villus.** Left: cells are labelled according to their ancestor cell; each crypt gives rise to a monoclonal population, with a multiclonal villus comprised of cells from each crypt. Right: the same simulation, here with cells labelled according to Delta levels (non-dimensionalised); Delta-Notch patterning occurs due to a signalling model inside each cell, which depends on the activity of neighbouring cells, and is thought to lead to differentiation into secretory and absorbative cell types. The simulation runs from 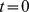 to 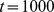 hours.(MP4)

Video S3
**Cardiac electrophysiology: a re-entrant spiral wave.** This figure displays the membrane voltage in a 2-D 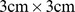 monodomain simulation using the Luo-Rudy 1991 action-potential model [50] with the modifications and protocol suggested in [49]. The simulation runs from 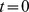 to 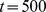 milliseconds.(MP4)

Video S4
**Cardiac electromechanics in a ventricular wedge: simulation of electrical propagation and deformation.** A stimulus is applied to the face 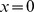 at 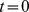 and the simulation runs until 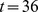 milliseconds.(MP4)
