# Supplementary material for: Chaste: An Open Source C++ Library for Computational Physiology and Biology
Source: PLoS Comput Biol. 2013 Mar 14;9(3):e1002970. doi: 10.1371/journal.pcbi.1002970 (PMC3597547; doi:10.1371/journal.pcbi.1002970)
Supplement: Text S1 — Further details on installation of Chaste and dependencies. (PDF) [file pcbi.1002970.s002.pdf]

Chaste: an open source C++ library for computational physiology and biology

Text S1: Supplementary Material

Supplementary material for the article consists of:

- This article, providing installation instructions and detail on Chaste dependencies.
- A Chaste 'project' download, also available at http://www.cs.ox.ac.uk/chaste/download in the "Bolt-on Projects" section.
- Video S1 — the simulation giving rise to Figure 1.
- Video S2 — the simulation giving rise to Figure 2.
- Video S3 — the simulation giving rise to Figure 3.
- Video S4 — the simulation giving rise to Figure 4.
- A series of wiki pages giving both the raw codes, and a tutorial-style guide, for reproducing the results that generated these figures at: https://chaste.cs.ox.ac.uk/trac/wiki/PaperTutorials/Plos2013.

1 Installation

In this section we summarise how to install and run Chaste. There are links to all the relevant information on the Chaste wiki page associated with this paper: https://chaste.cs.ox.ac.uk/trac/wiki/PaperTutorials/Plos2013.

Chaste must be run under a Linux environment, although this can consist of a Linux virtual machine, using software such as VirtualBox that is running on a Windows or Mac OS X system.[1]

As a C++ library, Chaste must be built on the computer system on which it is to be used. This requires downloading, building and installing its dependencies, and then Chaste itself. Users familiar with installing and using C++ libraries should not encounter any major difficulties. Those to whom

[1] We have begun work on a port to Microsoft Windows, but this is not available at release 3.1.

this is a new experience (this included many of the authors and core developers) can follow step-by-step guides, but should realise it can be a significant undertaking. For example:

- An Ubuntu dependency install should take on the order of 30 minutes, followed by a Chaste build of a few hours on a desktop PC (or around 30 minutes on an 8-core server).
- Other fresh manual Linux installations may take up to a day if none of the dependencies are set up.

We hope that the examples in the main paper will persuade you it may be worth the effort. Our mailing list community is happy to assist with any enquires about installing or building Chaste.

Ubuntu Install

The simplest installation method (by far) is to use the Ubuntu Linux operating system, as a 'one-click install' package is available for this platform. If you do not have Ubuntu installed then we recommend use of an Ubuntu virtual machine (e.g. via VirtualBox) on Windows and Mac OS X platforms, or to experiment with Chaste before committing to a full installation on a different Linux system. Up to date instructions for this can be found at `https://chaste.cs.ox.ac.uk/trac/wiki/InstallGuides/UbuntuPackage`.

Our policy is to support the newest Ubuntu release as soon as possible, along with all prior versions still supported by Ubuntu. Installation of Ubuntu 12.04 (LTS) therefore ensures compatibility with Chaste until at least 2017.

Other Linux systems

Chaste can also be installed on any other Linux system, via manual installation of a number of dependencies. Some scripts are provided on our wiki to simplify this process: instructions can be found via `https://chaste.cs.ox.ac.uk/trac/wiki/GettingStarted`.

Project for this article

A bolt-on project `Plos2013` has been made available that reproduces all of the examples in the article. It is available to download from the "Bolt-on projects" tab on the main Chaste download site: `http://www.cs.ox.ac.uk/chaste/download.html`.

2 Dependencies

The dependencies of Chaste are shown in Table 1. We aim to support a variety of versions of each library, to make installation simpler, as many of these are pre-installed on scientific computing services and HPC clusters. Our policy is to support those versions of the libraries that feature in the newest Ubuntu release, with all intermediate versions back to those in the oldest current long-term support editions of Ubuntu (this will include up to five years' worth of versions). A fine-grained list of compatible versions of the dependencies is provided at:

https://chaste.cs.ox.ac.uk/trac/wiki/InstallGuides/DependencyVersions

Table 1. Chaste Dependencies. For the full text of all licences examine the contents of `docs/licences.html` or visit https://chaste.cs.ox.ac.uk/trac/export/16530/trunk/docs/Licences.html. An asterisk indicates that the software is packaged with Chaste.

Dependency	Available from	Licence
Amara	http://wiki.xml3k.org/Amara	Apache 1.1
Boost	http://www.boost.org	Custom
CodeSynthesis XSD	http://codesynthesis.com/products/xsd	GPL
CVODE (SUNDIALS)	https://computation.llnl.gov/casc/sundials	BSD
CxxTest*	http://cxxtest.tigris.org	LGPL
HDF5	http://www.hdfgroup.org/HDF5	Custom
(Par)METIS	http://glaros.dtc.umn.edu/gkhome/views/metis	Custom
MPICH, or	http://www.mcs.anl.gov/research/projects/mpich2	Custom
Open MPI	http://www.open-mpi.org	BSD
PETSc	http://www.mcs.anl.gov/petsc	Custom
Pyparsing	http://pyparsing.wikispaces.com	MIT
RDFLib	https://github.com/RDFLib	BSD
RNV	http://www.davidashen.net/rnv.html	BSD
TetGen*	http://tetgen.berlios.de	Custom
Triangle*	http://www.cs.cmu.edu/~quake/triangle.html	Custom
VTK	http://www.vtk.org	BSD
Xerces	http://xerces.apache.org/xerces-c	Apache 2
